# Botulinum Toxin Injection into Epicardial Fat Pads: A Promising Potential Modality for Prevention of Postoperative Atrial Fibrillation After Cardiac Surgery

**DOI:** 10.21470/1678-9741-2019-0309

**Published:** 2019

**Authors:** Alireza Fatahian

**Affiliations:** 1 Department of Cardiology, Faculty of Medicine, Mazandaran University of Medical Sciences, Sari, Iran.

Despite developing new standards and advances in anesthetic and surgical techniques, postoperative atrial fibrillation (POAF) is one of the most frequent complications and a significant unsolved clinical problem after cardiac surgery^[1]^. Occurrence of POAF is associated with significant increased risk of long-term mortality, morbidity, and long intensive care unit (ICU) and hospital stays, which consequently impose additional cost on both health system and patient^[2]^. Considering the potential significant adverse effects of POAF in patients undergoing cardiac surgery and the limited efficacy of current preventive strategies, concerted efforts to identify and implement new preventive strategies are necessary^[1,2]^.

Recently, injection of botulinum toxin into epicardial fat pads in patients undergoing cardiac surgery has been suggested as a promising modality for prevention of POAF. In a study by Pokushalov et al.^[3]^, it has been shown that 50 U botulinum toxin injection, at four major epicardial fat pads, during surgery in patients undergoing coronary artery bypass grafting (CABG) provided considerable atrial tachyarrhythmia suppression, in both early and one-year follow-ups^[3]^. Also, a three-year follow-up of this study indicates a sustained and significant reduction in atrial fibrillation (AF) incidence and burden, along with reduction in patients' hospitalizations. Besides, no serious adverse events related to injection of botulinum toxin for prevention of POAF after CABG have been reported^[4]^. Suppression of atrial autonomic remodeling has been suggested as a potential mechanism of botulinum toxin injection for prevention of POAF^[3-4]^. Another study by Waldron et al.^[5]^ showed that injection of 50 U of botulinum toxin into each five epicardial fat pads (immediately after beginning of cardiopulmonary bypass) in patients undergoing cardiac surgery reduced the incidence of POAF compared to placebo (36.5% and 47.8%, respectively), however this difference was not statistically significant. Also, no significant differences were seen between length of hospital stay and occurrence of adverse effects in patients who received botulinum toxin injection or placebo^[5]^. In this study, patients who underwent CABG or valve surgery or combined CABG and valve surgery were included; whereas in Pokushalov et al.^[3]^ one-year and three-year follow-up studies^[4]^, only CABG patients were included and evaluated. Additionally, the left atrial sizes, as a marker of atrial structural remodeling, which commonly is associated with increased risk for AF, were considerably different between these studies (3.9±0.6 and 3.9±0.7 cm *vs*. 4.7±0.8 and 4.8±0.6, respectively). These issues may be possible explanations for the inconsistent results between studies. In sum, it seems that botulinum toxin injection into epicardial fat pads may be considered as a potentially promising and safe modality for prevention of POAF after cardiac surgery. However, further well-designed studies will be required to investigate the efficacy of this preventive modality for POAF in patients undergoing cardiac surgery, after controlling the potential confounding factors such as type of surgery, left atrial sizes, dose and site of injection of botulinum toxin, etc.
